# Whipple’s Disease Endocarditis Following Immunomodulatory Treatment for Arthritis: A Case Report and Screening Recommendation

**DOI:** 10.7759/cureus.67472

**Published:** 2024-08-22

**Authors:** Warren Back, James Meade, Salman Arif, Hend Elsaghir, Basmah Khalil, Claudiu Georgescu

**Affiliations:** 1 Medical School, The University of Toledo College of Medicine and Life Sciences, Toledo, USA; 2 Infectious Diseases, The University of Toledo College of Medicine and Life Sciences, Toledo, USA; 3 Internal Medicine, The University of Toledo College of Medicine and Life Sciences, Toledo, USA

**Keywords:** whipple’s disease, blood culture-negative endocarditis, endocarditis tropheryma whipplei, tropheryma whipplei, seronegative arthritis, tocilizumab, infective endocarditis

## Abstract

Whipple’s disease (WD) is a rare systemic disorder affecting various organ systems, including the gastrointestinal, cardiovascular, and joint systems. This report discusses a case of WD endocarditis likely associated with tocilizumab (TCZ), an immunomodulator used to treat refractory seronegative arthritis, in a patient with coronary artery disease and rheumatoid arthritis. The diagnosis was confirmed through laboratory studies, imaging, and esophagogastroduodenoscopy with biopsies. WD is increasingly recognized as a potential etiology of seronegative arthritis, with joint pain often preceding gastrointestinal symptoms. Immunomodulatory agents such as TCZ, while effective for rheumatoid arthritis, may exacerbate underlying WD, potentially leading to severe complications such as endocarditis. This case reveals the importance of considering WD in patients with refractory arthritis and the necessity of thorough diagnostic evaluation before initiating immunomodulatory therapy. Epidemiological studies indicate a higher prevalence of WD in certain demographics, highlighting the need for targeted screening with noninvasive screening methodologies, such as stool and saliva polymerase chain reaction testing.

## Introduction

Whipple’s disease (WD), first described by Dr. George Hoyt Whipple in 1907, is a rare systemic disorder caused by the gram-positive bacterium *Tropheryma whipplei*. This condition not only affects the gastrointestinal tract but also manifests in various organ systems, including the cardiovascular, central nervous, and joint systems. While initially characterized by malabsorption, arthralgias, and skin pigmentation, WD can present with diverse clinical features, making its diagnosis challenging. However, advancements in understanding its etiology, pathophysiology, and diagnostic criteria have improved over time, enabling more timely recognition and intervention. Although the gold standard for diagnosis remains a duodenal biopsy, the emergence of techniques such as stool and saliva polymerase chain reaction (PCR) has provided a noninvasive method for quick screening for this disease [1].

Despite its rarity, WD causes debilitating arthritis that is often refractory to traditional medications and even poses significant risks if left untreated. Culture-negative endocarditis is one such complication of untreated WD and has been associated with many negative outcomes, such as mycotic aneurysm [2], ischemic stroke [3], and embolic limb ischemia [4,5]. Further evidence suggests there may be a correlation between the use of immunomodulators, particularly tumor necrosis factor-alpha (TNF-α) inhibitors, and the incidence of endocarditis in WD patients [6-8]. Because immunomodulators, specifically tocilizumab (TCZ), are commonly used to treat refractory seronegative arthritis, it is imperative to consider the implication of worsening an underlying chronic *Tropheryma whipplei* infection [9]. Although the mechanism is not fully understood, it has been postulated that interleukin 6 inhibitors, such as TCZ, may mediate similar immune changes to TNF-α inhibitors, increasing susceptibility to worsening WD.

Here, we present a case of WD endocarditis and its likely association with TCZ, supporting the previously published literature.

## Case presentation

A 62-year-old Caucasian male with a past medical history of coronary artery disease with ST-elevation myocardial infarction status post left anterior descending artery stent placement and rheumatoid arthritis presented with worsening nausea, decreased appetite, joint pain, and generalized weakness. These symptoms had been present for two years and led to 25 pounds of unintentional weight loss. A review of systems was otherwise negative for fevers, chills, headache, chest pain, shortness of breath, vomiting, diarrhea, abdominal pain, urinary changes, muscle aches or pain, bleeding, bruising, numbness, and tingling. Despite current treatment with methotrexate and prednisone, the previously controlled joint pain was also worsening. He had been treated with TCZ injections every two weeks over a two-year period, with the last dose three months prior.

Social history revealed that he worked as an electrician, previously owned cats and dogs, grew up on a farm, enjoyed gardening and fishing, did not use tobacco, and his only travel included a trip to Nicaragua many years ago. A thorough physical examination was unremarkable with normal vital signs.

Initial laboratory studies had some mild abnormalities, including low sodium, calcium, protein, and hemoglobin levels, with mild elevations in glucose, alkaline phosphatase, and C-reactive protein (Table 1).

**Table 1:** Abnormal laboratory studies. CMP = complete metabolic panel; CBC = complete blood count; CRP = C-reactive protein

Lab parameter	Value	Reference range
CMP		
Sodium	134 mmol/L	136–145 mmol/L
Calcium	7.8 mg/dL	8.6–10.3 mg/dL
Glucose	124 mg/dL	70–100 mg/dL
Alkaline phosphatase	153 IU/L	34–104 IU/L
Total protein	4.8 g/dL	6.0–8.3 g/dL
Albumin	2.8 g/dL	3.5–5.7 g/dL
CBC		
Hemoglobin	11.1 g/dL	12.0–15.0 g/dL
CRP	38.4 mg/L	0.0–7.0 mg/L

A computed tomography scan of the chest was performed and demonstrated a small chronic, lobulated left pleural effusion with an interval decrease in size compared to one year prior. Transthoracic echocardiogram (TTE) showed normal functioning of the heart with a 0.9 × 0.877 cm aortic valve vegetation. Blood cultures were negative for growth, suggesting culture-negative endocarditis.

**Figure 1:**
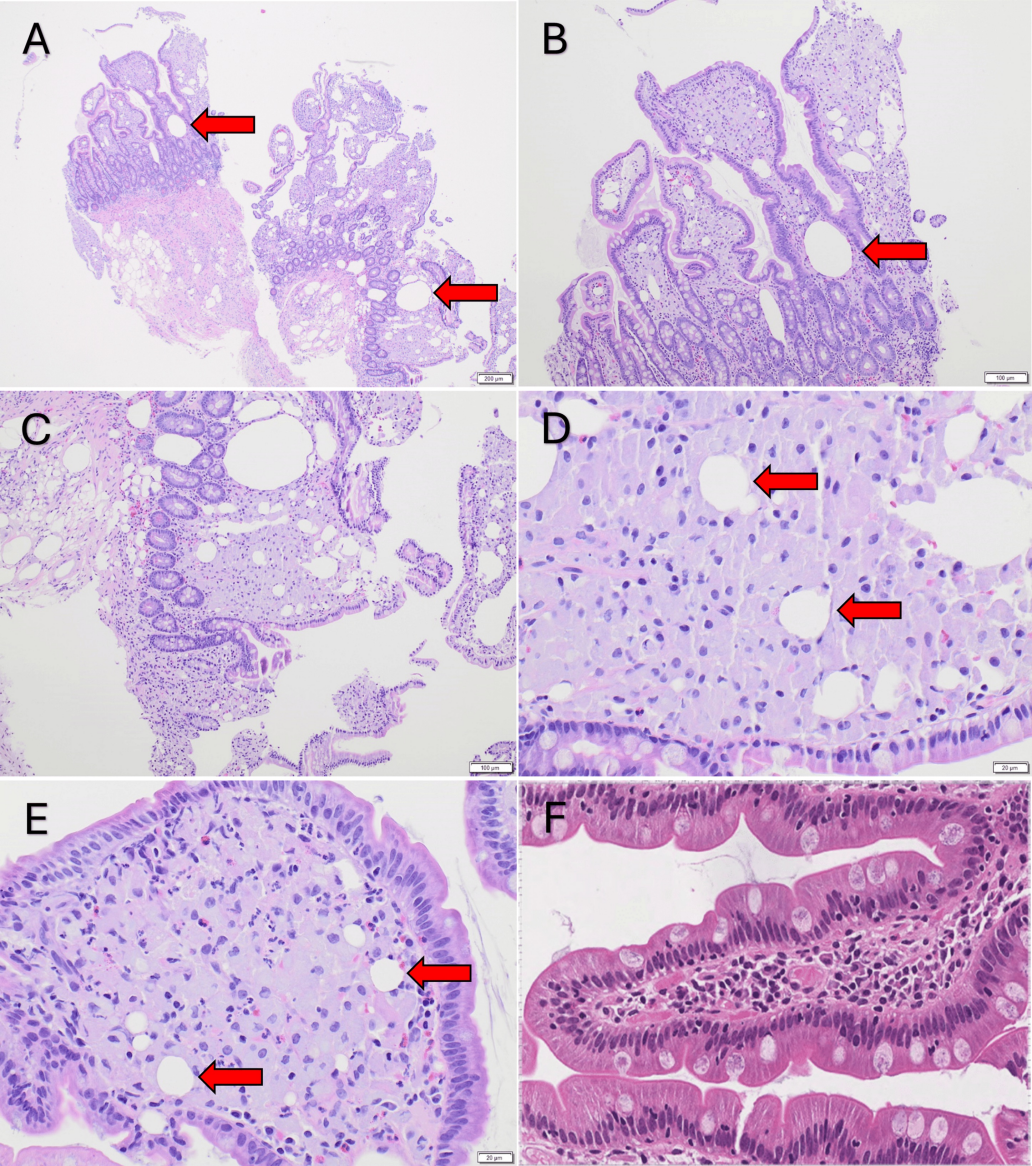
H&E stain of duodenal tissue biopsy showing foamy pink macrophages (red arrows) associated with lymphangiectasia. Increasing magnification of the same sample in each panel from A-E. Panel F demonstrates normal duodenal histology. H&E = hematoxylin and eosin

**Figure 2:**
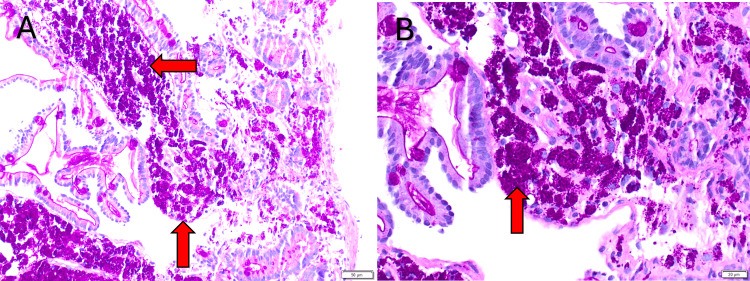
PAS with diastase stain of duodenal biopsy showing PAS-positive, diastase-resistant rod- and sickle-shaped bacterial inclusions (red arrows). Panel B is magnified. PAS = periodic acid-Schiff

**Figure 3:**
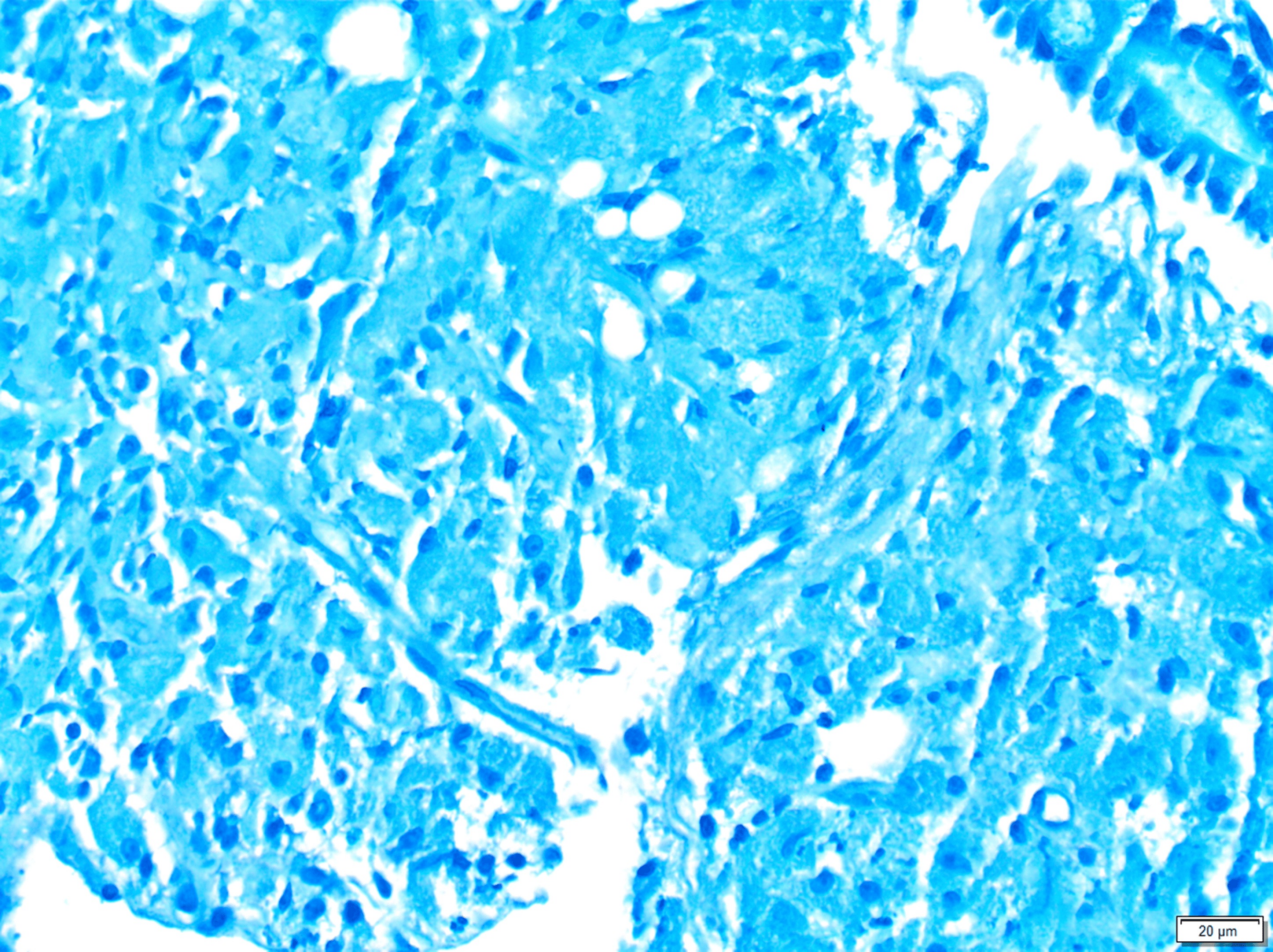
AFB stain of duodenal biopsy, negative for mycobacterial organisms. AFB = acid-fast bacilli

Further workup included an esophagogastroduodenoscopy (EGD) that revealed a normal esophagus, stomach, and duodenal bulb. A Z-line irregularity was observed at the gastroesophageal (GE) junction, as were thickened folds and scalloped mucosa in the duodenum, findings that were suggestive of celiac disease. Biopsies were obtained from the GE junction, stomach, and second and third parts of the duodenum. Immunohistochemistry staining with hematoxylin and eosin (H&E) (Figure 1), periodic acid-Schiff (PAS) with diastase (Figure 2), and acid-fast bacilli (Figure 3) was performed on the duodenal segments. The GE junction had hyperplastic squamous mucosa without metaplasia or dysplasia and the gastric biopsy was unremarkable. This confirmed the diagnosis of WD while also ruling out *Mycobacterium* infection. A tissue biopsy was sent for PCR testing, which returned positive for *Tropheryma whipplei*.

**Figure 4:**
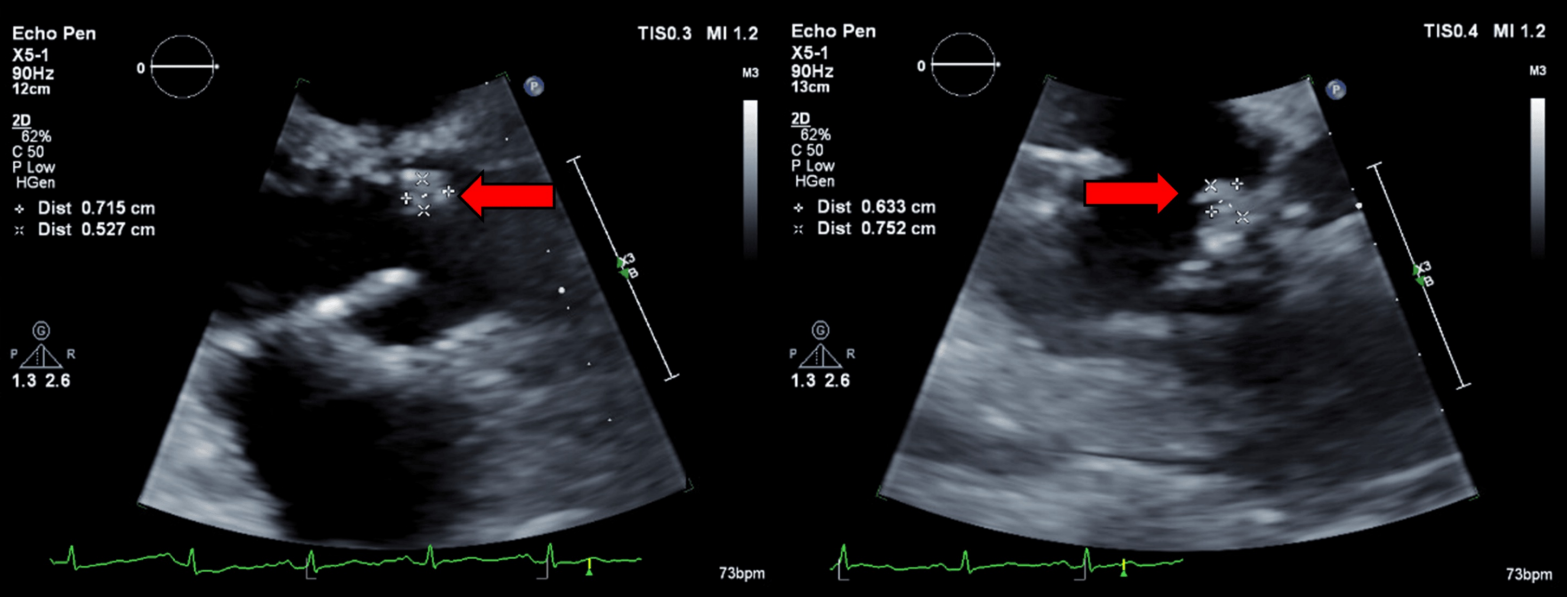
Repeat TTE redemonstrating aortic valve vegetation (red arrows) with measurements. TTE = transthoracic echocardiogram

The patient was then started on intravenous ceftriaxone 2 g daily for four weeks through a peripherally inserted central catheter. This was followed by oral trimethoprim-sulfamethoxazole 160/800 mg twice daily, which was discontinued shortly after due to abdominal pain. He was resumed on ceftriaxone at 4 g daily, double the original dosage. Repeat TTE three months later demonstrated an improved 0.715 × 0.527 cm mass (Figure 4). Transesophageal echocardiogram after one more month showed a 0.959 × 0.832 cm rounded echogenic density on the right coronary cusp of the aortic valve. He underwent aortic valve replacement due to severe aortic regurgitation seven months after the initial presentation. A pale tan to yellow, smooth, rubbery tissue mass measuring 1.6 × 0.7 × 0.9 cm was removed from the aortic valve. Following the procedure, antibiotics were switched to oral doxycycline 100 mg twice daily and oral hydroxychloroquine 200 mg three times daily for an indeterminate length of time.

EGD and biopsies of the stomach and duodenum five months later (one year after the initial presentation) were negative for signs of WD.

## Discussion

WD has been increasingly recognized as a potential etiology of seronegative arthritis, and while gastrointestinal manifestations are a hallmark, recent studies indicate that joint pain can precede them by several years [10]. DeBoer et al. reported the case of an 85-year-old Caucasian male who, much like our patient, presented with refractory arthritis and absent gastrointestinal symptoms and was later found to have WD [10]. This case underscores the importance of considering WD in patients with seronegative arthritis, particularly when classic gastrointestinal symptoms are absent. The complexity of diagnosing WD in such cases emphasizes the necessity for heightened clinical suspicion and thorough diagnostic evaluation, especially when standard arthritis therapies fail to yield improvement.

Immunomodulatory agents play a pivotal role in the treatment of various forms of arthritis by targeting inflammatory pathways. TCZ, a monoclonal antibody against the interleukin 6 receptor, has demonstrated efficacy in reducing disease activity and ameliorating symptoms in rheumatoid arthritis patients [8]. The use of immunomodulators necessitates careful consideration, particularly in individuals with underlying conditions such as WD. These agents have been shown to possibly increase the extracellular accumulation of *Tropheryma whipplei* in cardiac tissue, possibly predisposing to dissemination [6].

Multiple cases have been published demonstrating comorbidity of WD and culture-negative endocarditis [2-14]. Many of these patients were receiving immunomodulatory therapy, with the most common being TCZ [3,6-10]. Reports have even highlighted instances where immunomodulatory therapies, including monoclonal antibodies and TNF-α blockers, inadvertently exacerbated WD, leading to severe complications such as endocarditis [6-8]. Notably, the use of immunomodulators in combination with traditional antimicrobial therapies was reviewed in WD patients with comorbid endocarditis. Despite therapeutic interventions, mortality rates remained significant, demonstrating the complexity of managing WD-associated endocarditis [15].

In light of the potential exacerbation of WD by immunomodulatory therapies, a proactive approach involving screening for WD before initiating such treatments is warranted, particularly in patients with refractory arthritis. Epidemiological studies have revealed a higher prevalence of WD in certain demographics, such as Caucasians and individuals over 65 years old, emphasizing the need for targeted screening in these susceptible populations [16]. Although there was no difference in the prevalence of WD between sexes, males account for 85% of WD-endocarditis cases [16,17]. Clinicians should maintain a high index of suspicion for WD, especially in patients with refractory arthritis, and consider pre-emptive testing strategies to mitigate the risk of exacerbating underlying infections before initiating immunomodulatory treatment.

A similar approach is maintained regarding latent tuberculosis testing before beginning anti-TNF-α therapy for rheumatic diseases. The American Thoracic Society, United States Centers for Disease Control and Prevention, and Infectious Diseases Society of America issued guidelines in 2017 that classified patients as high-risk or low-risk for reactivation of latent tuberculosis and gave screening recommendations accordingly. Patients starting anti-TNF-α therapy were classified as high-risk, and it was recommended to use the tuberculin skin test, interferon-gamma release assay, or both to rule out tuberculosis before beginning the regimen [18]. Because administering immunosuppressants to patients with undiagnosed WD can similarly result in accelerated disease progression and comorbid conditions such as endocarditis, an analogous screening process should be adopted for WD.

Comparing other microbial causes of seronegative arthritis, such as Lyme disease (LD), supports the necessity of screening for WD. LD is well known for its late-manifesting arthritis that, much like WD, is refractory to the standard joint pain regimens [19]. What differentiates these two etiologies is the responsiveness to proper antibiotic therapy. The arthritis seen in LD can last years after eradication of the bacteria, and, in some cases, never fully dissipates [19]. In contrast, when WD is properly treated, there is a rapid reduction in joint pain leading to complete resolution [20]. This responsiveness to treatment reinforces the benefits of screening for WD in refractory seronegative arthritis patients because identification of the disease can lead to proper treatment and dramatic improvement in the patient’s quality of life.

The gold standard approach for diagnosis of WD is duodenal biopsy with H&E and PAS staining[1]. Because this is an invasive approach, we do not recommend implementing it as the first step unless EGD is already planned for other reasons. Instead, we recommend considering stool, saliva, and/or joint fluid PCR testing. Herbette et al. suggested a similar approach for WD screening in unexplained seronegative arthritis and showed that the combination of stool, saliva, and joint PCR has a negative predictive value of 98.5% in patients without constitutional or specific symptoms [21]. Another study by Fenollar et al. concluded that the combination of negative stool and saliva PCR alone has a negative predictive value greater than 99% for classic WD and suggested these be the mainstay for noninvasive screening [22]. These noninvasive screening methodologies, given negative results, should provide assurance to safely proceed with immunomodulatory therapy in patients with refractory arthritis. Positive screening results, however, would necessitate further workup with EGD and biopsy evaluation for definitive diagnosis before initiation.

## Conclusions

The case presented here illuminates the interplay between WD, refractory arthritis, and the potential exacerbating effects of immunomodulatory therapies. Despite its rarity, WD can present diverse clinical manifestations, often challenging clinicians to arrive at a timely diagnosis. The association between WD and culture-negative endocarditis, particularly in the context of immunomodulator use, highlights the importance of thorough diagnostic evaluation and consideration of underlying infections before initiating such treatments. As illustrated by this case and supported by existing literature, a proactive approach involving targeted screening for WD in high-risk populations, especially those with refractory arthritis, could be a crucial step in preventing severe complications such as endocarditis. By maintaining a heightened clinical suspicion and implementing pre-emptive testing strategies, clinicians can mitigate the risks associated with immunomodulatory therapies and ensure more effective management of WD-associated conditions.
